# Computed tomography imaging features of major pulmonary and extrapulmonary complications of fibrotic lung diseases

**DOI:** 10.31744/einstein_journal/2026RW0987

**Published:** 2025-12-01

**Authors:** Pedro Paulo Teixeira e Silva Torres, Paula Terra Martins Almeida Amaral, Alan Diniz Hummel, Eduardo Kaiser Ururahy Nunes Fonseca, Rodrigo Caruso Chate, Gilberto Szarf, Flávia Castro Velasco, Dante Luiz Escuissato, Marcelo Fouad Rabahi, Edson Marchiori

**Affiliations:** 1 Hospital Israelita Albert Einstein Goiânia GO Brazil Hospital Israelita Albert Einstein, Goiânia, GO, Brazil.; 2 Hospital Israelita Albert Einstein São Paulo SP Brazil Hospital Israelita Albert Einstein, São Paulo, SP, Brazil.; 3 Universidade Federal de Goiás Goiânia GO Brazil Universidade Federal de Goiás, Goiânia, GO, Brazil.; 4 Universidade Federal do Paraná Curitiba PR Brazil Universidade Federal do Paraná, Curitiba, PR, Brazil.; 5 Universidade Federal do Rio de Janeiro Rio de Janeiro RJ Brazil Universidade Federal do Rio de Janeiro, Rio de Janeiro, RJ, Brazil.

**Keywords:** Lung diseases, interstitial, Tomography, X-ray computed, Diagnostic imaging, Acute lung injury, Idiopathic interstitial pneumonias

## Abstract

Patients diagnosed with fibrosing interstitial lung disease are at risk of several complications, some of which may be life-threatening. Computed tomography imaging plays an important role in diagnosing these overlapping conditions. This article summarizes the computed tomography imaging features of the most common conditions associated with fibrosing interstitial lung diseases, categorized by involvement of the lung parenchyma or extra-pulmonary compartments. Some steps may help to recognize such complications, such as having knowledge of the underlying fibrotic lung disease, being aware of potentially immunosuppressive treatments in use, noting new relevant symptoms, checking previous imaging examinations to detect subtle changes, and considering the exam technique, for example, to avoid false perception of ground-glass opacities in exams with insufficient inspiration. Unenhanced computed tomography may be enough to diagnose most situations, but in specific cases, for example, in the clinical suspicion of pulmonary embolism, dedicated computed tomography angiography may be warranted. Careful comparison with previous exams is advised, especially to detect subtle opacities suspicious for lung cancer, underscoring that its detection may be difficult owing to the baseline morphological lung changes. Radiologists must be aware of such possible complications to perform early diagnosis and ensure proper management.

## INTRODUCTION

Imaging methods, especially computed tomography (CT), play important roles in the management of fibrosing interstitial lung disease (FILD), including disease detection (in both the preclinical and clinically established phases), identification of morphological patterns, determination of morphological behaviour and detection of complications.^( 1 , 2 )^Regardless of its aetiology – for example, idiopathic, auto-immune, environmental or occupational) – FILD places patients at risk of superimposed diseases, many of which are life-threatening.^( 3 , 4 )^ When considering such complications, clinicians and radiologists should ideally have knowledge of the underlying fibrotic lung disease, for example idiopathic pulmonary fibrosis (IPF), fibrotic hypersensitivity pneumonitis (FHP), collagen vascular disease (CVD), or occupational exposure; be aware of potentially immunosuppressive treatments in use; note new symptoms not associated with the baseline condition; consult previous imaging examinations for comparison to detect relevant new findings; and consider the examination technique, for example imaging acquisition during an insufficient inspiratory level, which may result in features mimicking new ground-glass opacity (GGO).

Examples of acute complications in patients with FILD include acute exacerbation (AE), infection, pulmonary embolism (PE), pneumothorax or pneumomediastinum, aspiration and hydrostatic oedema. Chronic disease that may complicate FILD include cancer, chronic infections (such as tuberculosis, saprophytic aspergillosis), dendriform pulmonary ossification (DPO) and pulmonary hypertension (PH).^( 1 , 5 , 6 )^

Computed tomography examination is indicated when FILD deterioration is clinically suspected; however, the optimal interval for routine follow-up examination has not been defined. In patients with systemic sclerosis, repeat CT examination at 12–24-month intervals (using a low-dose protocol to minimise radiation exposure) has been suggested to help assess progression and potentially influence prognosis.^( 7 , 8 )^ Recent guidelines for the management of IPF and progressive pulmonary fibrosis recommend annual CT examination to screen for complications – especially lung cancer (LC) – and to monitor the disease course. Unenhanced CT is the preferred examination for most evaluations, although the use of endovenous contrast may be warranted in certain cases, such as on the clinical suspicion of PE, as computed tomography pulmonary angiography (CTPA) protocols are accurate and widely available.^( 8 )^

In this article, we describe the imaging features of key parenchymal (pulmonary) and extraparenchymal complications of FILD ( Table 1 ).

**Table 1:** Summary of the major pulmonary complications of fibrosing interstitial lung disease, correlated with clinical and chest computed tomography findings

Complication	Clinical considerations	Chest CT findings
AE	IPF and those rheumatoid arthritis patients with UIP pattern on HRCT are at higher risk	New bilateral alveolar opacities are not attributable to hydrostatic oedema
Infection	Immunosuppressive therapy increases the risk of atypical and opportunistic infections; anti–tumour necrosis factor-α drugs may increase incidence of granulomatous lung infections	*Pneumocystis jirovecii* pneumonia: new heterogeneous GGO, crazy paving, eventually cysts
		Post-primary tuberculosis: new lung cavities, bronchogenic dissemination signs (centrilobular opacities), and possibly a higher frequency of lower lobe involvement
		Disseminated granulomatous infection (mycobacterial, fungal, other) may present with new miliary lung micronodules
	Corticosteroid use may increase the risk of superimposed aspergillosis	Aspergillomas: growing nodules or masses inside honeycombing cysts, mobile in decubitus; chronic pulmonary consolidations and cavitations with variable associated fibrosis
LC	Higher frequencies in patients with IPF, CV-ILD, fibrosis with emphysema, and a UIP pattern	Growing nodule or irregular opacity; special attention to lower lobe peripheral changes with UIP pattern
DPO	More frequent in patients with IPF, older age, male sex, and a history of smoking	Small calcified nodules, eventually branching, superimposed on reticular abnormalities
Extra-parenchymal		
Pneumomediastinum	May negatively affect prognosis; attention should be given to patients with inflammatory myopathies, especially those with anti-MDA5 positivity, as they may present with rapidly progressive ILD and pneumomediastinum	Abnormal gas collection in mediastinum
Pneumothorax	Patients with ILD and PPFE pattern are at risk, which may have a negative prognostic impact	Abnormal gas collection in pleural space
PE	Patients with IPF, HP and CV-ILD are at greater risk	Partial or complete filling defects in arterial branches may be detected by CTPA; attention should also be given to indirect findings (pulmonary infarction, hyperattenuating content inside pulmonary arteries), as many patients are examined using unenhanced chest CT
PH	Frequent in patients with FILD, especially in those with severe underlying parenchymal fibrosis	Increased pulmonary artery diameter, pulmonary artery/aorta ratio; right heart chamber enlargement; interventricular septal flattening

AE: acute exacerbations; CT: computed tomography; GGO: ground glass opacities; LC: lung cancer; IPF: idiopathic pulmonary fibrosis, CV-ILD: collagen vascular related interstitial lung disease; UIP: usual interstitial pattern; DPO: dendriform pulmonary ossification; anti-MDA5: anti–melanoma differentiation–associated gene 5 ; ILD – interstitial lung disease; PPFE: pleuroparenchymal fibroelastosis; PE: pulmonary embolism; HP: hypersensitivity pneumonitis; CTPA: computed tomography pulmonary angiography; FILD: fibrosing interstitial lung disease; PH: pulmonary hypertension.

## PARENCHYMAL COMPLICATIONS

### Acute exacerbation

**Figure 1:**
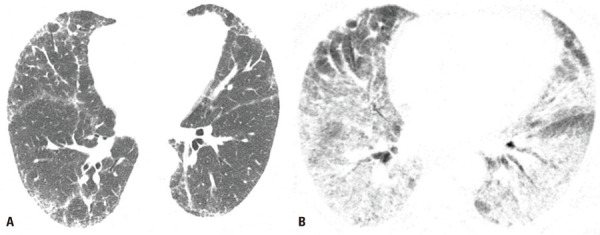
A 66-year-old man with familial pulmonary fibrosis. Axial chest computed tomography images obtained (A) at the time of diagnosis, showing predominantly peripheral fibrosing interstitial lung disease; and (B) during an acute exacerbation episode, showing new bilateral ground glass opacities

**Figure 2:**
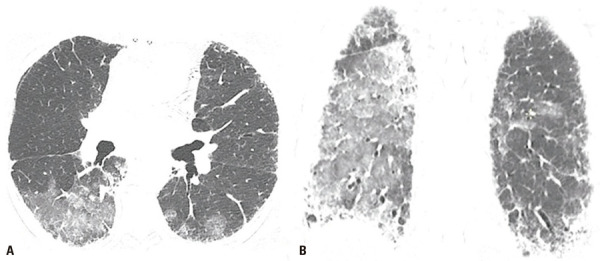
A 73-year-old man with fibrosing interstitial lung disease of unknown aetiology, who reported severe vomiting 2 days before the examination. Axial (A) and coronal reformatted (B) chest computed tomography images show new bilateral ground glass opacities attributable to exacerbation triggered by gastric content aspiration

**Figure 3:**
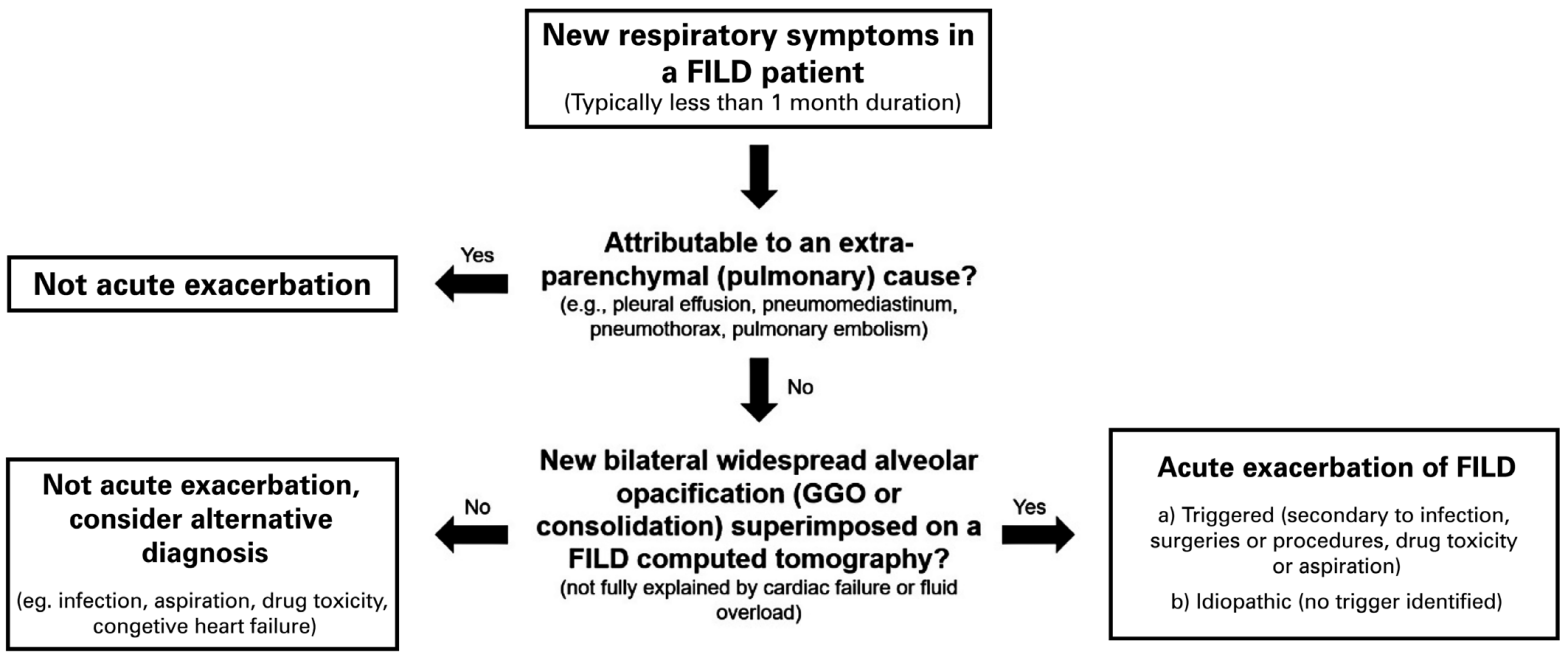
Diagnostic algorithm for fibrosing interstitial lung disease patients presenting with new symptoms

Adapted from the approach suggested for patients with idiopathic pulmonary fibrosis respiratory deterioration, as described by by Collard HR, Ryerson CJ, Corte TJ, Jenkins G, Kondoh Y, Lederer DJ, et al. Acute Exacerbation of Idiopathic Pulmonary Fibrosis. An International Working Group Report. Am J Respir Crit Care Med. 2016;194(3):265-75.^(^ ^3^ ^)^

FILD: fibrosing interstitial lung disease; GGO: ground-glass opacity.

Acute exacerbation is an unpredictable and potentially life-threatening complication of FILD, and the frequency of its occurrence is not precisely known.^( 3 , 4 )^ The IPF criteria for acute respiratory deterioration, have been described following the conceptual framework of Collard et al.,^( 3 )^ for patients presenting with dyspnoea (and cough, increased sputum production, fever and flu-like symptoms), with a somewhat arbitrary but typical interval of <1 month.^( 3 , 9 )^ Acute exacerbation should be assumed to have occurred in patients without evidence of an extra-parenchymal condition causing decompensation, who present with new bilateral lung opacities (such as GGO and consolidations) on CT that are not fully explained by cardiac failure or fluid overload. Without prior chest CT examinations for comparison, the presence of extensive alveolar opacities superimposed on a background of IPF is sufficient imaging evidence of AE ( Figure 1 ).^( 3 , 9 )^ Acute exacerbation is classified as idiopathic (when no trigger is identified) or triggered (such as by documented lung infection, post-procedurally or post-operatively, by drug use or even aspiration; Figure 2 ). This algorithm is intended for the diagnosis of IPF deterioration; however, given the similarity of exacerbations in patients with and without IPF and the lack of specific recommendations for the latter, some authors have suggested that it can also be applied to the detection of non-IPF FILD exacerbation ( Figure 3 ).^( 4 , 9 )^

Causes of acute deterioration not meeting the criteria for AE include pneumothorax, pleural effusion, PE, congestive heart failure, infection, aspiration and drug toxicity. On chest CT, predominant findings such as septal thickening, opacity with a central distribution, pleural effusion, and eventually cardiomegaly should favour the diagnosis of hydrostatic pulmonary oedema over inflammatory AE.^( 10 , 11 )^

Acute exacerbation episodes have been examined within the framework of acute lung injury.^( 3 )^ Histologically, the main pattern implicated is diffuse alveolar damage in the acute or exudative and organising stages. Other patterns, such as organising pneumonia, acute fibrinous and organising pneumonia, alveolar haemorrhage and other nonspecific inflammatory changes may be superimposed on various fibrotic patterns (for example UIP, non-specific interstitial pneumonia (NSIP) associated with the underlying condition.^( 3 , 9 , 12 , 13 )^

**Figure 4:**
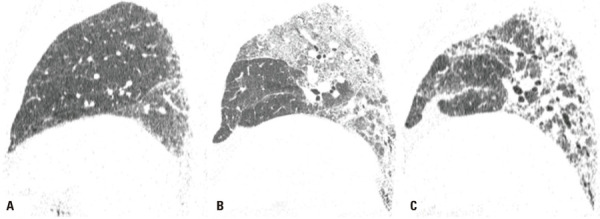
A 75-year-old man with familial pulmonary fibrosis. Chest computed tomography images with sagittal reformatting were obtained (A) at the time of diagnosis, showing predominantly peripheral and basal reticular opacities; (B) during an acute exacerbation, showing new extensive ground-glass opacities; and (C) on follow-up, showing significant progression of fibrotic lung abnormality and pulmonary volume loss

Akira et al.^( 14 )^ showed that the extent and distribution of new lung opacities representing AE on CT correlated with survival in patients with IPF, with a diffuse distribution being a stronger predictor of mortality than a predominantly peripheral or multifocal distribution. Kato et al.^( 15 )^ also found that a diffuse pattern of CT involvement was most common in fatal episodes of AE. Other studies confirm that the disease extent on CT correlates with the outcome.^( 16 , 17 )^ In AE survivors, the new opacities may either resolve or progress to fibrosis, with denser foci of consolidation and increasing traction bronchiectasis ( Figure 4 ).^( 14 )^

The baseline CT pattern of FILD also appears to be associated with the risk of AE. Kang et al.^( 18 )^ found that a UIP-like pattern (meeting the compatible and probable UIP criteria of the Fleischner Society IPF guidelines^( 19 )^) together with reduced lung diffusing capacity for carbon monoxide, was a risk factor for AE in patients with HP. Hozumi et al.^( 20 )^ also recognised the UIP pattern on CT as a risk factor for AE in patients with rheumatoid arthritis. In patients aged >80 years who had received long-term oxygen therapy before hospital admission, the UIP pattern also increases the risk of death from AE.^( 21 )^ Among FILD aetiologies, IPF and asbestosis are associated with the shortest AE survival time, whereas NSIP is linked to the most favourable AE prognosis.^( 21 )^

### Infection

Infection can trigger AE in patients with and without IPF, and its diagnosis may be challenging.^( 22 )^ Survival time is reported to be longer and mortality lower for infection-triggered AE than for AE of other aetiologies (idiopathic and triggered by other factors).^( 15 )^ The frequency of viral detection in FILD patients during AE (both IPF and non-IPF) is highly variable in literature, ranging from 9–48%.^( 23 - 26 )^ Several viruses can be detected by multiplex polymerase chain reaction (PCR) platforms in AE of FILD, including human herpesvirus 7, cytomegalovirus, influenza virus, human parainfluenza virus, human metapneumovirus, coronavirus, respiratory syncytial virus and others.^( 23 - 26 )^

Among patients with 2019 coronavirus disease (COVID-19), pre-existing ILD increases the odds of worse outcomes (death, hospitalisation and intensive care unit-level care) and reduces the likelihood of discharge compared with patients without ILD.^( 27 )^ COVID-19 may also trigger AE of ILD.^( 27 , 28 )^ Esposito et al.^( 28 )^ reported that the UIP pattern was more frequent in COVID-19 non-survivors than in survivors, although the difference was not significantly.

Infectious diseases contribute substantially to the morbidity and mortality of patients with CVD. Multi-drug and immunosuppressive treatments may increase the risk of atypical and opportunistic infections, including *Pneumocystis jirovecii* pneumonia, in these patients.^( 4 , 22 , 29 - 31 )^ On CT, features of opportunistic infection, drug toxicity and interstitial complications may be difficult to differentiate from those of underlying CVD.^( 30 )^

**Figure 5:**
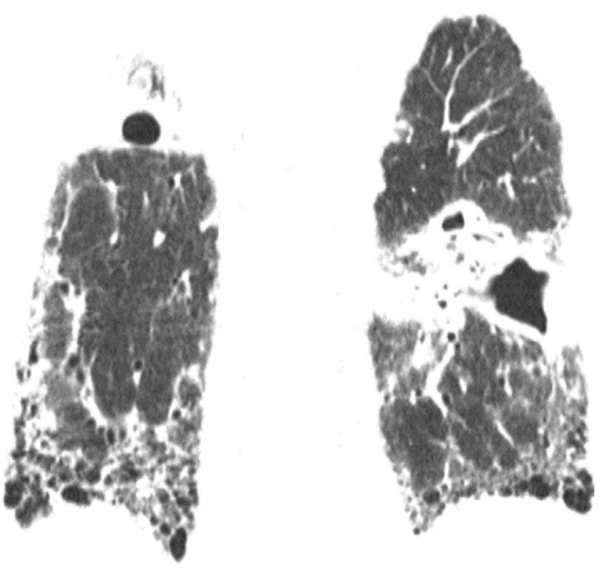
A 62-year-old man with fibrosing interstitial lung disease of unknown aetiology and a usual interstitial pneumonia pattern. Chest computed tomography image shows consolidation and a cavity in the superior segment of the left lower lobe, diagnosed as pulmonary tuberculosis

Biological agents, such as anti-tumour necrosis factor-α drugs, are used to treat several autoimmune diseases, and their pathophysiological effects to granuloma formation (particularly those of monoclonal antibodies such as infliximab) increase the risk of mycobacterial and fungal granulomatous infection.^( 31 )^ In these patients (using biological agents), tuberculosis is usually secondary to reactivation and new fungal infections (for example, histoplasmosis, coccidioidomycosis, blastomycosis) may be acquired acutely. The clinical presentation of tuberculosis in these cases often resembles that in immunocompetent hosts; however, >50% of patients present with disseminated disease.^( 31 )^ Together with laboratory tests, chest x-ray and CT may be useful for screening latent tuberculosis; in positive cases, chemoprophylaxis before immunosuppressive treatment is recommended.^( 31 , 32 )^ Owing to structural lung abnormalities in patients with FILD, CT findings attributable to tuberculosis may be difficult to detect. Except for distribution, the imaging findings in these patients are similar to those in patients with active tuberculosis without FILD and include consolidation, cavities and pulmonary nodules. A greater frequency of lower lobe involvement has been reported for the UIP and non-UIP patterns ( Figure 5 ).^( 33 , 34 )^

**Figure 6:**
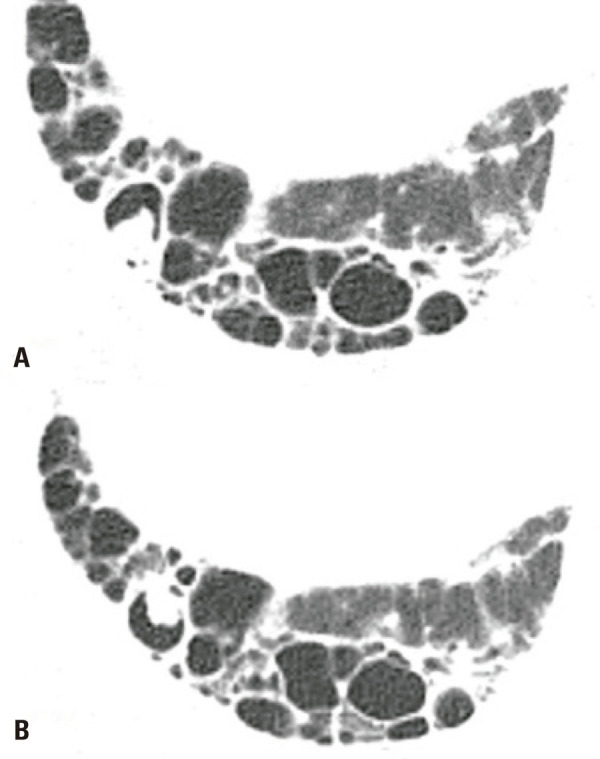
A 52-year-old female with a usual UIP due to rheumatoid arthritis. Chest computed tomography images show a small pulmonary nodule inside a honeycombing cyst (A), mobile on decubitus (B), attributable to chronic pulmonary aspergillosis

In a retrospective cohort of patients with ILDs of various aetiologies, those with chronic pulmonary aspergillosis (CPA) defined by serum anti- *Aspergillus* antibody positivity, were on average older, predominantly male, and has smoking histories; 34% of these patients had IPF.^( 35 )^ In the same cohort, the majority of patients with FILD and CPA showed the UIP pattern with honeycombing, smoking-related ILD, and upper lobe predominance on CT.^( 35 )^ Corticosteroid use is a major risk factor for CPA and its progression of dissemination.^( 35 )^ On CT, CPA may appear as new or growing cavities with variable wall thickening, mainly in the upper lobes, along with adjacent lung opacities and bronchiectasis, and pleural thickening or effusion. In some cases these findings are association with consolidations showing central necrosis and cavity formation.^( 36 )^ The detection of saprophytic aspergillosis (presenting as nodules inside honeycombing cysts) on chest CT, is particularly important for differential diagnosis with LC. A nodule or mass inside a pre-existing cavity or honeycombing cyst that is mobile on prone complementary ventral decubitus acquisition should favour CPA ( Figure 6 ).^( 37 )^

### Lung cancer

Patients with ILD are at greater risk of developing LC than the general population.^( 38 )^ Reported prevalence of LC in European and Asian FILD cohorts are 11.6% and 15.3%, respectively.^( 39 )^ The risk of LC is 3.34 times higher in patients with IPF, 2.3 times higher in those without IPF and 4.95 times higher in those with CV-ILD, compared to the general population^( 40 )^ LC is more prevalent in patients with IPF and emphysema with the UIP pattern than in those with IPF alone.^( 41 )^ The presence of FILD *,* such as IPF, may have important negative prognostic implications in patients with LC.^( 42 )^ In one study, the median time from IPF diagnosis to LC development was 16.3 months.^( 43 )^

**Figure 7:**
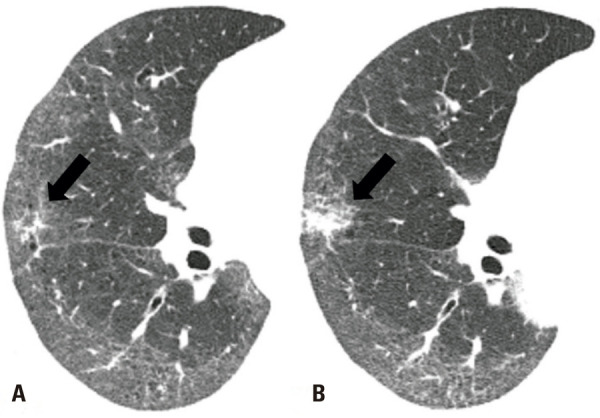
A 69-year-old woman with a fibrotic NSIP pattern owing to systemic sclerosis. Axial chest computed tomography images show a subtle irregular opacity in the right middle lobe (arrow in A), which increased in size at the 1-year follow-up (arrow in B) and was diagnosed as squamous cell carcinoma

Most LC lesions in patients with FILD are associated with fibrotic regions and show peripheral lower-lobe predominance, although presentation may vary.^( 38 , 44 )^ Among 547 surgically resected cases of LC, the majority of patients with a UIP pattern had squamous cell carcinoma with peripheral lower-lobe predominance, whereas most patients with non-UIP patterns had adenocarcinoma, most commonly affecting the upper lobes.^( 45 )^ A systematic review and meta-analysis showed that LC in patients with IPF was most frequently peripheral and involved, in descending order, the right lower, left lower, right upper, and left upper lobes.^( 46 )^ The presence of fibrotic lung changes can make the differential diagnosis between focal fibrosis and potential LC challenging ( Figure 7 ); careful comparison with previous examinations and positron emission tomography or CT examination may be useful in such cases.^( 43 )^ Given the peripheral predominance of LC in this context, CT-guided percutaneous transthoracic needle biopsy (PTNB) may be a feasible option for the diagnostic procedure.^( 47 )^ Shin et al.^( 48 )^ found that PTNB performs reasonably well in diagnosing LC in patients with IPF (89% accuracy, 90% sensitivity and 84% specificity); however, the complications rate is high (50% overall and 12% for major complications). Factors associated with non-diagnostic results included lesion size <3cm and needle tip placement outside the target lesion, and the presence of honeycombing along the needle pathway was an independent risk for major complications.^( 48 )^ In that study, AE represented 2% of the complications. Kim et al.^( 49 )^ found that acquiring two or fewer biopsy samples was a risk factor for non-diagnostic PTNB results in patients with a UIP pattern, and that using a long needle pathway was a risk factor for pneumothorax development.

The presence of FILD must be considered in patients undergoing surgical or systemic LC treatment. Chemotherapy can induce potentially fatal AE in 5-20% of patients with ILD.^( 47 )^ Ground-glass attenuation and increased fibrosis on chest CT are risk factors for the development of pneumonitis in patients with pre-existing ILD or interstitial lung abnormalities (ILAs) receiving immune checkpoint inhibitors; close monitoring is warranted in these cases.^( 47 )^ Pulmonary and non-pulmonary surgical interventions are risk factors for ILD AE, which occurs in 9-23% of patients after surgical resection of LC.^( 47 )^

Lung cancer screening programmes may provide opportunities for the early diagnosis of ILD, enabling early management and treatment. In one cohort undergoing low-dose CT screening for LC, LC was detected in 2.5% of patients, ILA affecting >5% of the lung were detected in 4.2%, and 1.51% of patients met the criteria for ILD.^( 50 )^

### Dendriform pulmonary ossification

**Figure 8:**
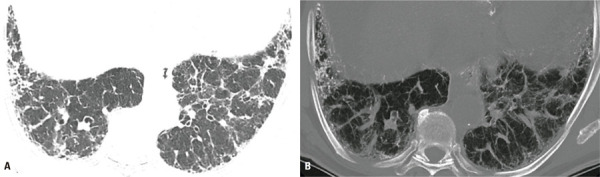
A 70-year-old man with idiopathic pulmonary fibrosis. (A) Axial chest computed tomography image showing fibrosing interstitial lung disease with a probable usual interstitial pneumonia pattern, and (B) Window-adapted maximum intensity projection showing small clustered and branching calcified nodules attributable to dendriform pulmonary ossification

Dendriform pulmonary ossification, characterized by the spread of small ossified pulmonary nodules through the lung parenchyma, is uncommon and may be idiopathic or associated with a variety of conditions, including cardiac diseases, severe lung injury and FILD. In a cohort of patients with FILD, DPO was significantly associated with IPF and not with other aetiologies, such as NSIP and FHP.^( 51 )^ Risk factors for DPO included advanced age, male sex, smoking history, and CT features such as honeycombing, emphysema and a coarser interstitial pattern, all characteristics strongly associated with IPF.^( 51 )^ On CT, DPO appears as small calcified pulmonary nodules that eventually branch, predominantly superimposed on subpleural fibrotic areas ( Figure 8 ).^( 51 )^The recently updated diagnostic criteria for IPF note that a UIP pattern is associated with the possibility of pulmonary ossification.^( 8 )^

## EXTRA-PARENCHYMAL COMPLICATIONS

### Pneumomediastinum and pneumothorax

**Figure 9:**
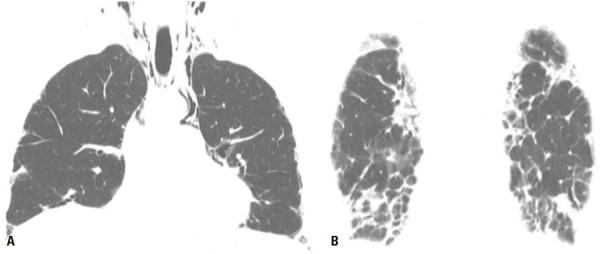
A 40-year-old man with amyotrophic dermatomyositis (anti–melanoma differentiation–associated gene 5 positive). (A) Coronal chest computed tomography image showing pneumomediastium with cervical extension, and (B) Reticular and perilobular peripheral lung opacities indicating interstitial lung disease with a chronic organizing pneumonia pattern

Pneumomediastinum, defined as the presence of air in the mediastinal ( Figure 9 ), may complicate ILD. Its most common clinical manifestation is new or worsening dyspnoea, and it may co-exist with pneumothorax.^( 52 )^ The condition most commonly develops through rupture of alveoli adjacent to connective tissue and blood vessels, allowing air to enter the perivascular space and migrate along the bronchovascular sheath to the mediastinal soft tissues.^( 53 , 54 )^ In patients with ILD, the worsening of parenchymal abnormalities may be associated with pneumomediastinum, and the use of steroids may increase lung frailty, thereby raising the risk of this complication.^( 52 )^ In patients with IPF and those with CVD, pneumomediastinum, particularly when persistent,is associated with poor prognosis.^( 52 )^ Pneumomediastinum is prevalent in patients with dermatomyositis or polymyositis, conditions often complicated by ILD; certain subtypes are associated with rapidly or potentially progressive interstitial lung involvement, which in turn is associated with anti–melanoma differentiation–associated gene 5 (MDA5) positivity.^( 52 , 55 )^ Spontaneous pneumomediastinum in patients with this condition can worsen prognosis, increasing mortality rate to 60%.^( 56 )^ In a series of hospitalised patients with dermatomyositis or polymyositis, spontaneous pneumomediastinum occurred more frequently in anti-MDA5-positive patients and its occurrence increased the mortality risk.^( 57 )^ In these patients, pneumomediastinum is closely linked to rapidly progressive ILD, reflecting the severity of pulmonary involvement.^( 55 )^

**Figure 10:**
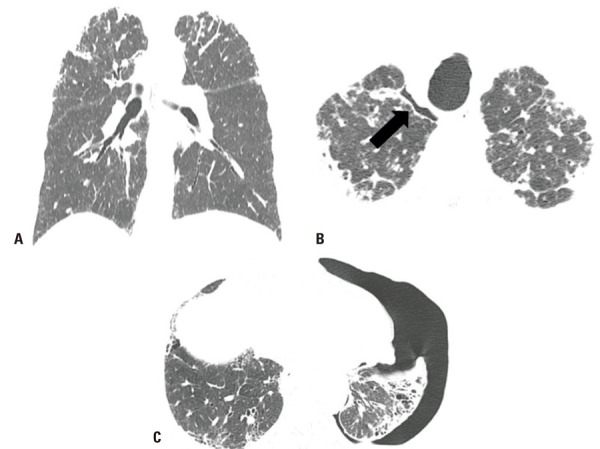
A 65-year-old man with fibrosing interstitial lung disease of unknown aetiology. Coronal chest computed tomography images show apical reticular opacities with pleuroparenchymal fibroelstosis features (A) and a small pneumothorax (arrow in B). (C) A 60-year-old man with a usual interstitial pattern of unknown aetiology presenting with moderate left pneumothorax

Pneumothorax and pneumomediastinum are frequent complications of ILD with a PPFE pattern on CT ( Figure 10 ). For example, pneumothorax was at least three times more common among patients on a lung transplant waitlist with ILD with a PPFE pattern than among those without this pattern.^( 58 )^ Failure of pneumothorax to resolve despite intervention may have prognostic relevance in these patients. In patients with IPF, the cumulative incidence of pneumothorax over 3 years was 17.7%, and this condition was identified as an independent predictor of poor outcome.^( 59 )^ PPFE is seen in 6-10% of patients with IPF and may be associated not only with increased risks of pneumothorax and pneumomediastinum, but also with a more rapid decline in lung function and poorer survival rate.^( 8 , 60 )^

### Pulmonary embolism

Idiopathic pulmonary fibrosis can increase the risk of venous thromboembolism (VTE) two-fold compared to the general population, although the exact mechanism involved is unknown.^( 61 )^ Possible explanations are a) the greater inflammatory burden, b) fibrogenic cytokine induction by thrombin (a key enzyme in the coagulation cascade), c) immobility due to respiratory symptoms and reduced exercise capacity and d) the use of glucocorticoids to treat AE.^( 61 )^

Fibrosing interstitial lung disease of other aetiologies is also associated with an increased risk of VTE. In patient with IPF, risk factors for VTE include systemic steroid treatment and advanced disease stage, whereas in patient with FHP, arterial hypertension and PH are risk factors; the incidence of VTE was reported to be similar in the two groups.^( 62 )^ In a retrospective cohort of patients with ILD, the prevalence of PE was 14.6%, and the most frequently associated conditions were IPF, CVD and FHP.^( 63 )^

**Figure 11:**
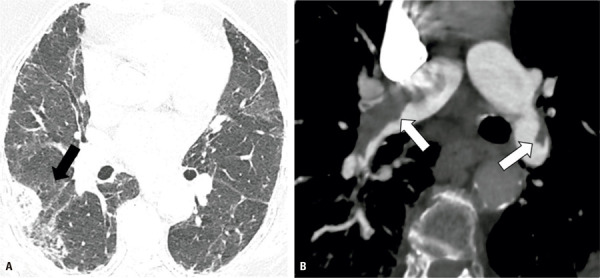
An 84-year-old woman with fibrosing interstitial lung disease of unknown aetiology and worsening dyspnoea. (A) Axial chest computed tomography image showing a new peripheral consolidation with a reversed halo morphology, suspicious of pulmonary infarction. (B) Computed tomography pulmonary angiography obtained subsequently, showing extensive filling defects in the main pulmonary arteries

Computed tomography pulmonary angiography enables accurate detection of PE and should be considered in some clinically decompensated patients or when indirect findings of PE, such as peripheral consolidations with a reversed halo morphology or even spontaneous hyperattenuating content within pulmonary arteries and branches, are observed ( Figure 11 ).^( 64 )^

### Pulmonary hypertension

Idiopathic pulmonary fibrosis, CVD and FHP, among other FILDs, may be complicated by PH. Pulmonary hypertension has been reported in up to 86% of ILD cases, especially in patients with severe underlying parenchymal fibrosis, and is associated with poor prognosis. Given its negative impact on prognosis, PH screening is recommended for patients with ILD.^( 65 )^

Complementary examinations may aid the diagnosis and follow-up of PH in patients with FILD. These include pulmonary function and exercise tests, echocardiography, imaging (chest radiography, chest CT, ventilation-perfusion lung scintigraphy and magnetic resonance imaging) and hemodynamic assessment, the last being especially recommended when PH may affect the management of the underlying ILD.^( 65 , 66 )^

**Figure 12:**
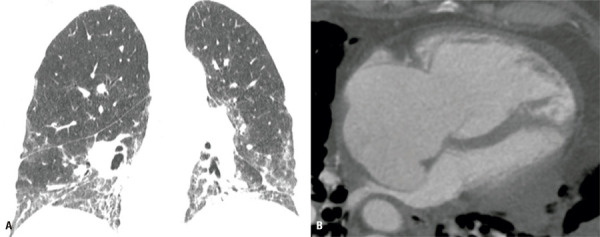
A 55-year-old man with rheumatoid arthritis. Axial chest computed tomography images show (A) ground glass and reticular opacities in a nonspecific interstitial pneumonia pattern, and (B) marked right chamber enlargement and leftward bowing of the interventricular septum due to pulmonary hypertension

Chest CT signs that may raise suspicion of PH include a) the transverse diameter of the pulmonary artery, b) a pulmonary artery/aorta ratio >1, c) right ventricular enlargement (right/left ventricle ratio >1) and d) flattening of the interventricular septum ( Figure 12 ).^( 65 )^ Several factors may affect the influence pulmonary artery diameter and pulmonary artery/aorta ratio; older age, male sex and larger body surface area are correlated with larger pulmonary arteries, and the pulmonary artery/aorta ratio decreases with age and should not be considered in the presence of aortic dilatation.^( 65 )^ Additionally, highly variable CT-measured pulmonary artery diameter cut-off values for PH suspicion have been reported. In patients at high risk of PH, including those with FILD, PH may occur at any pulmonary artery transverse diameter or pulmonary artery/aorta ratio, and normal CT values should not be used to rule out this diagnosis.^( 66 )^

## CONCLUSION

Patients with fibrosing interstitial lung disease are at risk of developing several complications, some of which adversely affect prognosis, and computed tomography may contribute significantly to the diagnosis and management of these complications. As many of these patients have distorted lung architecture and a myriad of fibrotic lung findings on computed tomography, careful attention should be paid to any subtle new or growing opacity to avoid delaying the diagnoses of important conditions, such as lung cancer and chronic granulomatous infectious disease. Radiologists must also be aware of complications that occur more frequently with specific conditions or treatments and should employ optimal examination techniques along with careful comparison to previous examinations to facilitate early diagnosis.

DATA AVAILABILITY:

The underlying content is contained within the manuscript.
